# Supplementary material for: Oxidative Stress-Mediated Antibacterial Activity of the Total Flavonoid Extracted from the Agrimonia pilosa Ledeb. in Methicillin-Resistant Staphylococcus aureus (MRSA)
Source: Vet Sci. 2022 Feb 7;9(2):71. doi: 10.3390/vetsci9020071 (PMC8874552; doi:10.3390/vetsci9020071)
Supplement: Supplementary file 1 [file vetsci-09-00071-s001.zip › vetsci-1569120-supplementary.pdf]

veterinary sciences

MDPI

Article

Oxidative Stress-Mediated Antibacterial Activity of the Total Flavonoid Extracted from the *Agrimonia pilosa* Ledeb. in Methicillin-Resistant *Staphylococcus aureus* (MRSA)

Liren He [1], Han Cheng [1], Fuxin Chen [2], Suquan Song [1], Hang Zhang [3], Weidong Sun [1], Xiaowei Bao [4], Haibin Zhang [1] and Chenghua He [1,*]

[1] College of Veterinary Medicine, Nanjing Agricultural University, Nanjing 210095, China; 2019107090@njau.edu.cn (L.H.); chenghan@stu.njau.edu.cn (H.C.); suquan.song@njau.edu.cn (S.S.); swd100@njau.edu.cn (W.S.); haibinzh@njau.edu.cn (H.Z.)

[2] School of Chemistry and Chemical Engineering, Xi'an University of Science and Technology, Xi'an 710054, China; chenfuxin@xust.edu.cn

[3] Key Laboratory of Advanced Drug Preparation Technologies, Ministry of Education, Co-Innovation Center of Henan Province for New Drug R & D and Preclinical Safety, School of Pharmaceutical Sciences, Zhengzhou University, Zhengzhou 450001, China; hangzhang@zzu.edu.cn

[4] College of Food Science and Pharmacy, Xinjiang Agricultural University, Urumqi 830052, China; xiaoweibao0723@xjau.edu.cn

* Correspondence: hechenghua@njau.edu.cn; Tel.: +86-025-8439-5227; Fax: +86-025-8439-8669

Supplementary Materials

Table S1. The list of flavonoid compounds extracted from the *A. pilosa* Ledeb.

Peak	Parent ion	MS/MS	Error (ppm)	Mol. formula	Proposed compound
1	271.0601	158.0122	1.366	$C_{15}H_{10}O_5$	apigenin
2	285.0395	229.0506, 151.0037	-3.132	$C_{15}H_{10}O_6$	kaempferol
3	287.0586	289.0596, 288.0582, 153.0167, 135.0424	-0.552	$C_{15}H_{10}O_6$	luteolin
4	301.0363	273.0413, 255.0304, 227.0363125.0242,	2.668	$C_{15}H_{12}O_7$	taxifolin
5	303.0499	301.0347, 273.0417, 302.0432	1.547	$C_{15}H_{10}O_7$	quercetin
6	431.1055	117.0341, 283.0633, 161.0241, 135.0437, 121.0288	-2.987	$C_{21}H_{20}O_{10}$	vitexin
7	449.1078	287.0553, 288.0586, 289.0614, 451.1134	0.658	$C_{21}H_{20}O_{11}$	kaempferol-3-*O*-glucoside
8	465.1028	303.0495, 304.0535, 301.0342, 305.0544	3.374	$C_{21}H_{20}O_{12}$	isoquercetin
9	593.1353	594.1394, 595.1412, 285.0425	4.636	$C_{30}H_{26}O_{13}$	tiliroside
10	610.1508	300.0263, 299.0184， 270.0162	1.517	$C_{27}H_{30}O_{16}$	Rutin

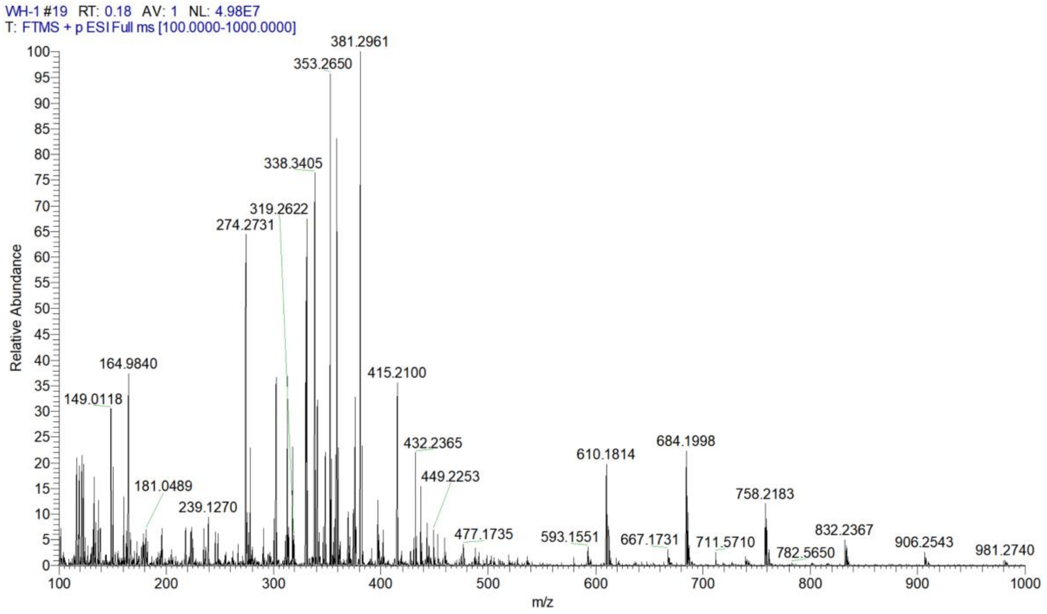

Figure S1. The total ion chromatogram of total flavonoid extracted from the *A. pilosa* Ledeb.

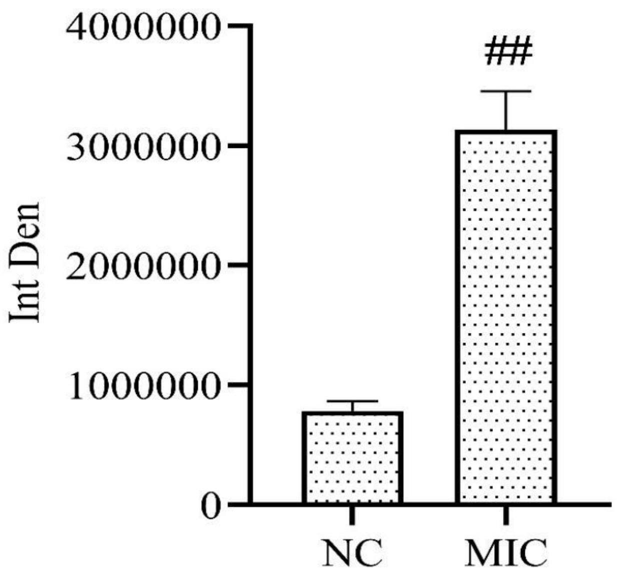

Figure S2. The fluorescence intensity of intracellular ROS in MRSA ATCC43300 analyzed by the Image J software. ## means p-value < 0.01.

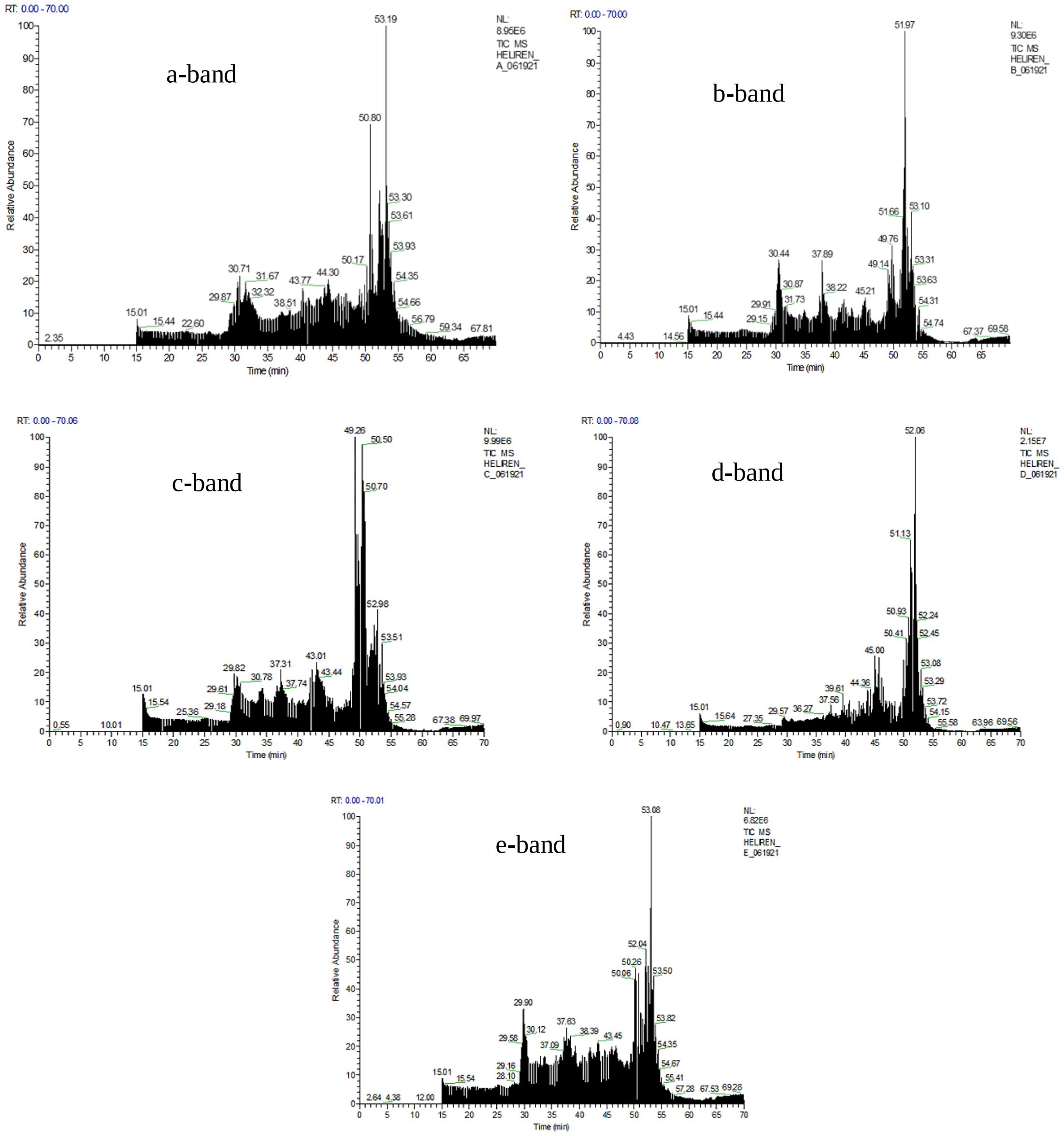

Figure S3. The total ion chromatogram of the a-band, b-band, c-band, d-band and e-band cut from the SDS-PAGE.

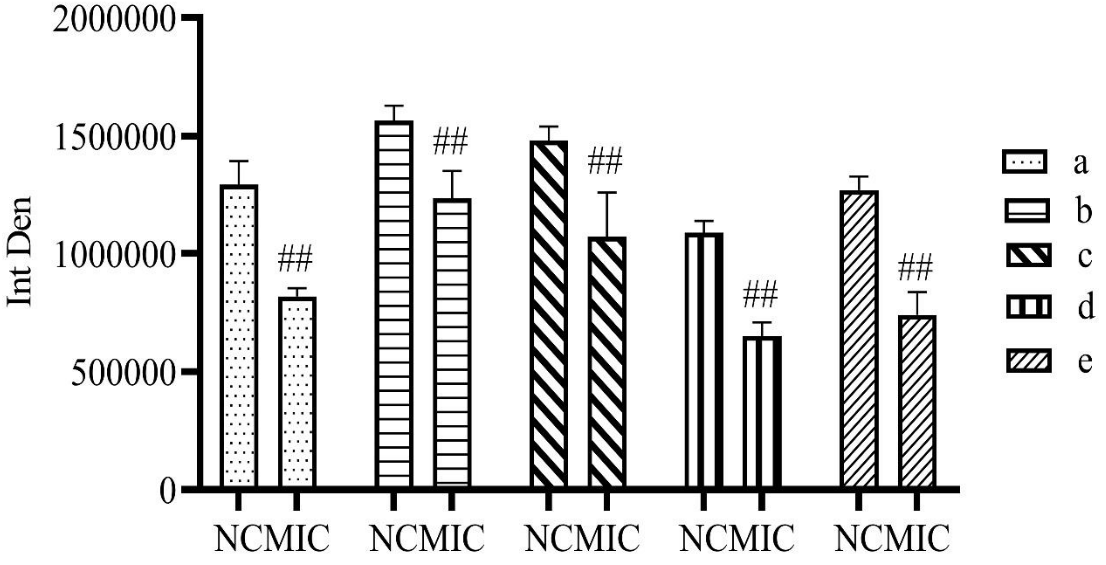

Figure S4. The densitometry analysis of a-band, b-band, c-band, d-band and e-band by the Image J software. ## means *p*-value < 0.01.
